# Pathogenic *Leptospira* Species in Insectivorous Bats, China, 2015

**DOI:** 10.3201/eid2406.171585

**Published:** 2018-06

**Authors:** Hui-Ju Han, Hong-Ling Wen, Jian-Wei Liu, Xiang-Rong Qin, Min Zhao, Li-Jun Wang, Li-Mei Luo, Chuan-Min Zhou, Ye-Lei Zhu, Rui Qi, Wen-Qian Li, Hao Yu, Xue-Jie Yu

**Affiliations:** Wuhan University, Wuhan, China (H.-J. Han, J.-W. Liu, X.-R. Qin, M. Zhao, L.-J. Wang, C.-M. Zhou, R. Qi, X.-J. Yu);; Shandong University, Jinan, China (H.-L. Wen, Y.-L. Zhu, W.-Q. Li);; Shandong Center for Disease Control and Prevention, Jinan (L.-M. Luo); Fudan University, Shanghai, China (H. Yu)

**Keywords:** Leptospira, bat, Eptesicus serotinus, Myotis fimbriatus, Myotis ricketti, Myotis pequinius, vector-borne infections, zoonoses, Mengyin, Shandong, China, rrs2, multilocus sequence typing, MLST, bacteria

## Abstract

PCR amplification of the *rrs2* gene indicated that 50% (62/124) of insectivorous bats from eastern China were infected with *Leptospira borgpetersenii*, *L. kirschneri*, and several potentially new *Leptospira* species. Multilocus sequence typing defined 3 novel sequence types in *L. kirschneri*, suggesting that bats are major carriers of *Leptospira*.

Leptospirosis is a zoonotic disease caused by the pathogenic spirochetes of the bacterial genus *Leptospira* (*1*). Although leptospirosis is mainly prevalent in tropical and subtropical countries (*2*), it is considered an emerging or reemerging zoonosis of global public health concern (*1*). In China, leptospirosis is listed as a category B notifiable disease (*3*). Globally, rodents are recognized as important reservoir hosts (*4*); however, a growing number of studies highlight the potential role of bats in the epidemiology of *Leptospira* (*4*). So far, knowledge on *Leptospira* in bats in China is lacking. Therefore, we screened archived bat kidney samples for *Leptospira* species to evaluate the potential role of bats in the ecology of *Leptospira* in China.

## The Study

During July–October 2015, we captured 124 insectivorous bats with nets in Mengyin County, Shandong Province, China; the bats were initially intended for viral metagenomic analysis. Details regarding anesthetization of bats and tissue sampling were described previously (*5*). We collected the kidneys, storing them at −80°C until analysis. We identified bat species by using PCR amplification and DNA sequencing of the cytochrome B (*cytB*) gene as described previously (*6*). The 124 bats were classified into 4 species of the Vespertilionidae family (26 *Eptesicus serotinus* bats from 2 farmers’ houses, 30 *Myotis fimbriatus* bats and 10 *M. ricketti* bats from a city sewer, and 58 *Myotis pequinius* bats from a karst cave).

We designated bat kidney samples by using the abbreviation of Shandong plus the sample identification number (e.g., SD-49). We extracted DNA from bat kidney samples by using QIAamp DNA Mini Kit (QIAGEN, Hilden, Germany), according to the manufacturer’s instructions. To identify the species of *Leptospira* in bats, we amplified the 16S rRNA gene (*rrs2*) by using nested PCR with primers Lepto 16S-1st-F, Lepto 16S-1st-R, Lepto 16S-2nd-F, and Lepto 16S-2nd-R (*7*). We cloned the *rrs2* PCR products (642 bp) into the pMD 19-T vectors (TaKaRa, Shiga, Japan) and randomly picked 1 colony for Sanger sequencing using M13 universal primers on both DNA strands.

On the basis of *rrs2* amplification results, 62 (50.0%) of 124 bats tested were positive for *Leptospira*. All the *E. serotinus* bats (0/26, 0.0%) were negative for *Leptospira*, whereas the *M. fimbriatus* bats (19/30 [63.3%]), *M. ricketti* bats (6/10 [60.0%]), and *M. pequinius* bats (37/58 [63.7%]) showed high rates of infection.

**Figure 1:**
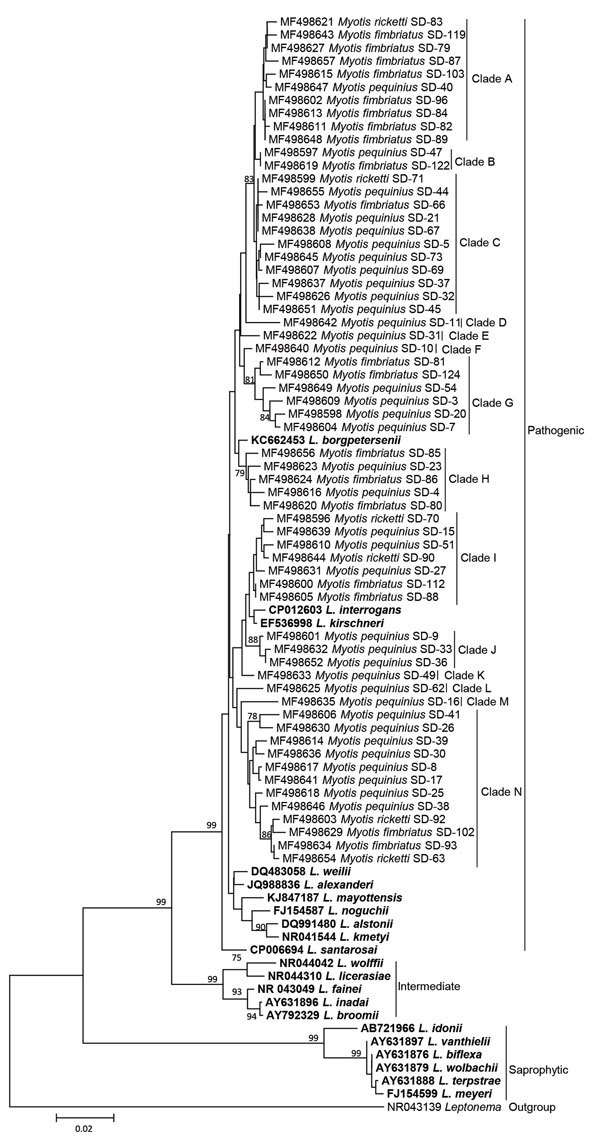
Neighbor-joining phylogenetic tree based on *rrs2* gene of *Leptospira* isolates from bats, Mengyin County, Shandong Province, China, and reference *Leptospira* sequences from GenBank (boldface). We constructed the tree with the *rrs2* sequences (642 bp) from this study by using the Kimura 2-parameter model with MEGA 7.0 (http://www.megasoftware.net); we calculated bootstrap values with 1,000 replicates. Sequences of *Leptospira* detected in bats in this study are shown with the GenBank accession number, the Latin name of the bat species in which *Leptospira* was detected, and the corresponding bat number. Only bootstrap values >75% are shown. Scale bar indicates nucleotide substitutions per site.

**Figure 2:**
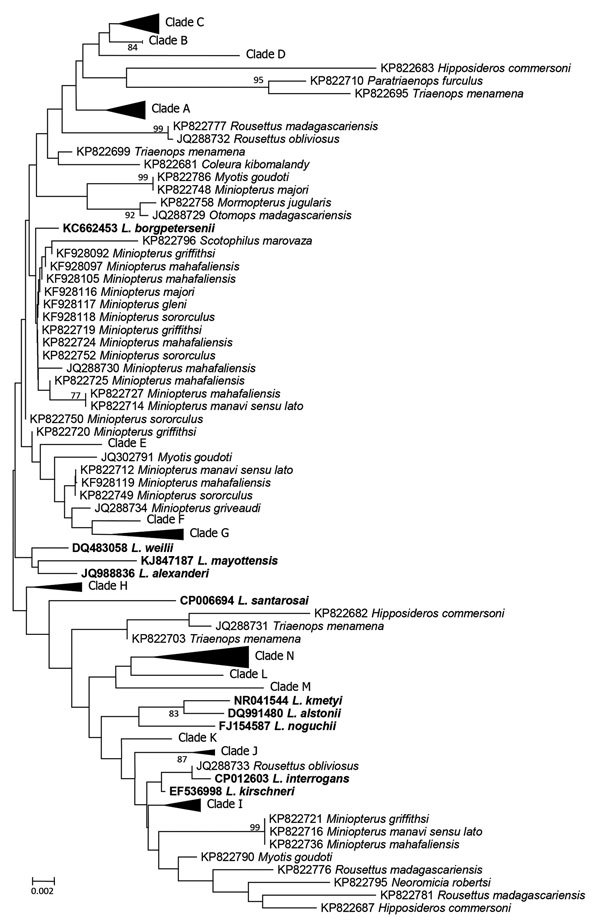
Neighbor-joining phylogenetic tree based on *rrs2* gene of pathogenic *Leptospira* isolates from bats, Mengyin County, Shandong Province, China, and reference *Leptospira* sequences from GenBank that had been previously isolated from bats (boldface). We constructed the tree with bat *Leptospira rrs2* sequences (446 bp) from this study and previous studies by using Kimura 2-parameter model with MEGA 7.0 (http://www.megasoftware.net); we calculated bootstrap values with 1,000 replicates. *Leptospira* detected in this study are shown with compressed clades, and bat *Leptospira* sequences from previous studies are shown with the GenBank accession number and the Latin name of the bat species in which *Leptospira* was detected. Only bootstrap values >75% are shown. Scale bar indicates nucleotide substitutions per site.

Using ClustalW with MEGA 7.0 (http://www.megasoftware.net), we aligned the *rrs2* sequences from this study with reference *Leptospira* species downloaded from GenBank. We constructed a neighbor-joining phylogenetic tree (also by using MEGA 7.0) (*8*). On the basis of the *rrs2* phylogeny, all *Leptospira* detected in bats in Mengyin County clustered into the pathogenic group and could be divided into >14 clades (clades A–N). Although clade H was most likely associated with *L. borgpetersenii*, the *Leptospira* detected in bats in Mengyin County were divergent from any known *Leptospira* species (Figure 1) and any previously published *Leptospira* sequences from bats (Figure 2); therefore, these organisms might represent potentially new *Leptospira* species. We deposited the 62 *rrs2* sequences of *Leptospira* in the Mengyin County bats into GenBank (accession nos. MF498596–657).

To characterize the *Leptospira* species detected in bats from Mengyin County, we attempted multilocus sequence typing (MLST) on 7 housekeeping genes (*glmU*, *pntA*, *sucA*, *tpiA*, *pfkB, mreA*, and *caiB*), as previously described (*9*). We assigned alleles for all 7 loci by using a publicly available *Leptospira* MLST website (https://pubmlst.org/leptospira) and defined sequence types (STs) by using the allelic profiles (*glmU*-*pntA*-*sucA*-*tpiA*-*pfkB*-*mreA*-*caiB*).

Because we conducted MLST using kidney DNA rather than DNA from isolates, the results were arduous to obtain. Only 35 of the 62 *rrs2*-positive bats were successfully amplified for >1 gene. For three samples, SD-49, SD-88, and SD-112, all 7 loci were obtained. We uploaded the allele data to the *Leptospira* MLST database and assigned a novel allele number for each gene (Technical Appendix Table). According to the allelic profiles, we classified the organisms as *L. kirschneri*, assigning all 3 with novel STs (ST244 for SD-49, ST246 for SD-88, and ST245 for SD112).

We obtained 32 incomplete allelic profiles in all. After searching the *Leptospira* MLST database for each gene, we found that the loci of this study could not match with any known alleles and that they represented novel alleles; however, novel alleles could not be assigned for incomplete allelic profiles. Phylogenies based on each of the 7 genes showed inconsistent topologies for individual bats, indicating co-infection with different *Leptospira* species in the Mengyin County bats (Technical Appendix Table, Figures 1–7). For SD-103, for example, we amplified 5 of the 7 genes. On the basis of *glmU*, *tpiA*, *pfkB*, and *mreA*, SD-103 clustered with *L. borgpetersenii*; however, on the basis of *sucA*, SD-103 fell into the group of the potentially new *Leptospira* sp. 2. 

Because we did not conduct culture isolation with the archived bat kidney samples, further isolation with fresh bat kidney or urine samples will be needed to enable a thorough genotyping analysis of *Leptospira* species in the Mengyin County bats. Altogether, our study suggested that bats in Mengyin County carried a wide diversity of *Leptospira*, and the actual genetic diversity is likely even higher.

In our study, all the *E. serotinus* bats were negative for *Leptospira*, whereas *M. fimbriatus*, *M. ricketti*, and *M. pequinius* bats showed a high rate of infection. This finding might be explained by a widely accepted belief that *Leptospira* mainly circulate in humid environments (*10*). *E. serotinus* bats were captured from the eaves of 2 farmers’ houses, where the habitats were dry, whereas the *M. fimbriatus*, *M. ricketti*, and *M. pequinius* bats were sampled from the humid city sewer and karst cave.

So far, *Leptospira* has been detected in ≈50 bat species belonging to 8 bat families from tropical and subtropical regions as well as part of Europe (*4*). Although the role of bats as carriers of *Leptospira* associated with human leptospirosis remains uncertain, intrusion into bat habitats and increasing urbanization that results in the cohabitation of bats and humans are likely to increase the opportunity for batborne *Leptospira* spillover (*11*). Moreover, bats might play an important role in the epidemiology of *Leptospira* through transmission between bats and rodents, with rodents being a major source of human infection (*12*).

## Conclusions

*Myotis* spp. bats in Mengyin County in Shandong Province of China showed a high rate of infection with *Leptospira borgpetersenii*, *L. kirschneri*, and several potentially new *Leptospira* species, suggesting that bats are important carriers of *Leptospira* in Mengyin County. To date, knowledge of batborne leptospirosis is lacking, and the monitoring of the potential spillover of batborne *Leptospira* to humans is needed.

Technical Appendix Multilocus sequence typing results and phylogenetic trees for each of the 7 housekeeping genes of *Leptospira* detected in bats, Mengyin County, Shandong Province, China.
