# Supplementary material for: Pathogenic Leptospira Species in Insectivorous Bats, China, 2015
Source: Emerg Infect Dis. 2018 Jun;24(6):1123–6. doi: 10.3201/eid2406.171585 (PMC6004874; doi:10.3201/eid2406.171585)
Supplement: Technical Appendix — Multilocus sequence typing results and phylogenetic trees for each of the 7 housekeeping genes of Leptospira detected in bats, Mengyin County, Shandong Province, China. [file 17-1585-Techapp-s1.pdf]

Article DOI: https://doi.org/10.3201/eid2406.171585

Pathogenic *Leptospira* Species in Insectivorous Bats, China, 2015

Technical Appendix

Technical Appendix Table. Multilocus sequence typing (MLST) of *Leptospira* in bats, Mengyin County, Shandong Province, China

Bat species	Bat no.	*glmU*	*pntA*	*sucA*	*tpiA*	*pfkB*	*mreA*	*caiB*	ST	Co-infection
M. fimbriatus	SD-88	*Lk* (62)	*Lk* (73)	*Lk* (68)	*Lk* (63)	*Lk* (88)	*Lk* (65)	*Lk* (60)	ST246	1
M. fimbriatus	SD-112	*Lk* (63)	*Lk* (74)	*Lk* (69)	*Lk* (64)	*Lk* (89)	*Lk* (66)	*Lk* (60)	ST245	1
M. fimbriatus	SD-124	*Lb*	—	*Lb*	—	—	—	—	—	1
M. fimbriatus	SD-122	N1	N1	N1	—	—	N1	—	—	1
M. fimbriatus	SD-93	N1	N1	N1	N1	N1	—	—	—	1
M. fimbriatus	SD-103	*Lb*	—	N2	*Lb*	*Lb*	*Lb*	—	—	2
M. fimbriatus	SD-102	N1	—	N1/N2	N1	N1/*Lb*	N1	—	—	3
M. ricketti	SD-92	N1	N1	N1/*Lb*	N1	N1	N1	—	—	2
M. pequinius	SD-49	*Lk* (61)	*Lk* (72)	*Lk* (67)	*Lk* (62)	*Lk* (87)	*Lk* (64)	*Lk* (59)	ST244	1
M. pequinius	SD-10	*Lk*	*Lk*	*Lk*	—	*Lk*	*Lk*	*Lk*	—	1
M. pequinius	SD-15	*Lk*	*Lk*	*Lk*	—	*Lk*	*Lk*	*Lk*	—	1
M. pequinius	SD-36	*Lk*	*Lk*	*Lk*	—	*Lk*	—	—	—	1
M. pequinius	SD-21	*Lb*	—	—	—	—	—	—	—	1
M. pequinius	SD-23	*Lb*	—	*Lb*	*Lb*	*Lb*	—	*Lb*	—	1
M. pequinius	SD-38	*Lb*	—	*Lb*	*Lb*	*Lb*	—	—	—	1
M. pequinius	SD-8	N1	N1	N1	—	—	N1	—	—	1
M. pequinius	SD-30	N1	N1	N1	—	N1	N1	—	—	1
M. pequinius	SD-62	N1	N1	N1	—	N1	N1	—	—	1
M. pequinius	SD-41	N1	N1	N1	N1	N1	—	—	—	1
M. pequinius	SD-39	N1	—	N1	—	N1	N1	—	—	1
M. pequinius	SD-25	*Lk*	*Lk*	*Lk*	—	*Lb*	—	*Lk*	—	2
M. pequinius	SD-9	*Lk*	*Lk*	*Lk*	*Lb*	*Lk*	*Lk*	*Lk*	—	2
M. pequinius	SD-27	*Lk*	*Lk*	*Lk*	*Lb*	*Lk*	*Lk*	*Lk*	—	2
M. pequinius	SD-3	*Lb*	—	*Lb*	—	N2	*Lb*	—	—	2
M. pequinius	SD-40	*Lb*	—	*Lb*	—	N2	*Lb*	—	—	2
M. pequinius	SD-5	*Lb*	N1	N1	—	*Lb*	—	—	—	2
M. pequinius	SD-69	*Lb*	N2	N2	—	*Lb*	*Lb*	—	—	2
M. pequinius	SD-33	*Lb*	*Lk*	N2	—	*Lb*	*Lb*	—	—	2
M. pequinius	SD-37	*Lb*	—	N2	—	*Lb*	*Lb*	—	—	2
M. pequinius	SD-45	*Lb*	—	N2	—	*Lb*	*Lb*	—	—	2
M. pequinius	SD-26	N1	*Lk*	N1	N1	N1	N1	*Lk*	—	2
M. pequinius	SD-31	*Lb*	N1	*Lb*	*Lb*	N2	N1	—	—	3
M. pequinius	SD-16	*Lb*	N1	*Lb*	—	N2	*Lb*	—	—	3
M. pequinius	SD-11	*Lb*	*Lk*	N4	—	—	—	*Lk*	—	3
M. pequinius	SD-17	N1	N1	N3	N1	N1	N1	*Lk*	—	3

*For SD-49, SD-88, and SD-112, novel alleles assigned are shown in the parentheses. *Lb*, *L. borgpetersenii*; *Lk*, *L. kirschneri*; N1, potential new *Leptospira* sp. 1; N2, potential new *Leptospira* sp. 2; N3, potential new *Leptospira* sp. 3; N4, potential new *Leptospira* sp.

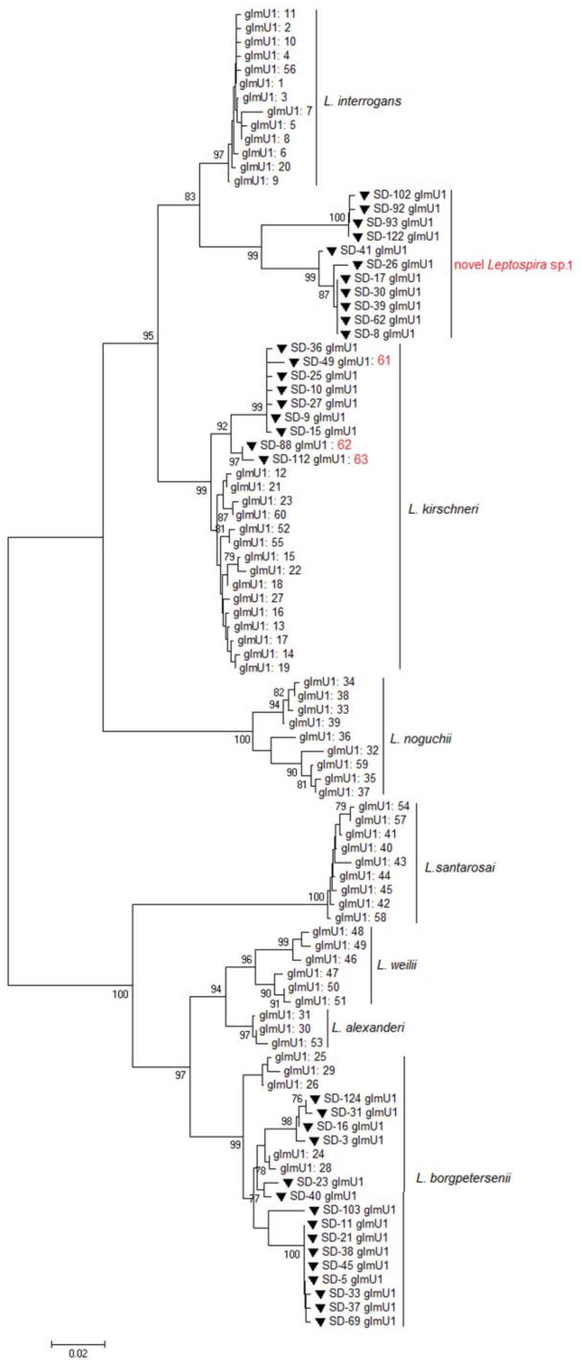

Technical Appendix Figure 1. Neighbor-joining phylogenetic tree for *glmU* gene of *Leptospira* in bats, Mengyin County, Shandong Province, China. The tree was constructed with Kimura's 2-parameter model using MEGA 7.0, and bootstrap values were calculated with 1,000 replicates. Alleles of each locus detected in this study are shown with the sample number highlighted with a triangle, and novel allele numbers assigned in this study are indicated in red. *Leptospira* species are shown on the right, and potential new *Leptospira* species detected in this study are shown in red.

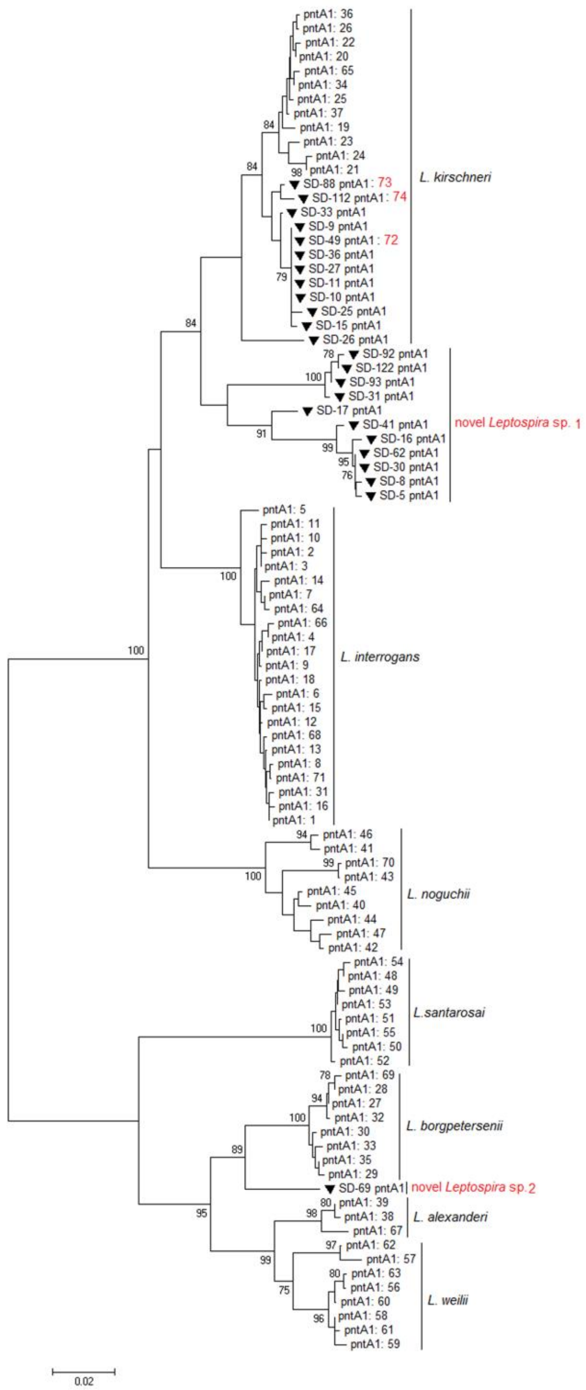

Technical Appendix Figure 2. Neighbor-joining phylogenetic tree for *pntA* gene of *Leptospira* in bats, Mengyin County, Shandong Province, China. The tree was constructed with Kimura's 2-parameter model using MEGA 7.0, and bootstrap values were calculated with 1,000 replicates. Alleles of each locus detected in this study are shown with the sample number highlighted with a triangle, and novel allele numbers assigned in this study are indicated in red. *Leptospira* species are shown on the right, and potential new *Leptospira* species detected in this study are shown in red.

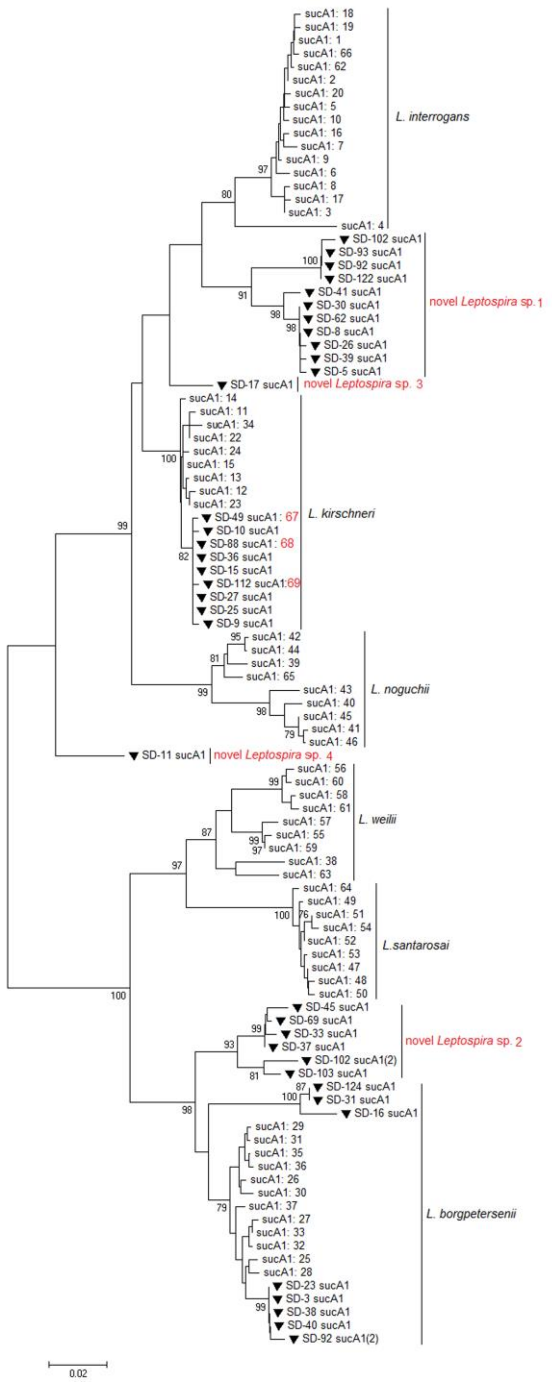

Technical Appendix Figure 3. Neighbor-joining phylogenetic tree for *sucA* gene of *Leptospira* in bats, Mengyin County, Shandong Province, China. The tree was constructed with Kimura's 2-parameter model using MEGA 7.0, and bootstrap values were calculated with 1,000 replicates. Alleles of each locus detected in this study are shown with the sample number highlighted with a triangle, and novel allele numbers assigned in this study are indicated in red. *Leptospira* species are shown on the right, and potential new *Leptospira* species detected in this study are shown in red.

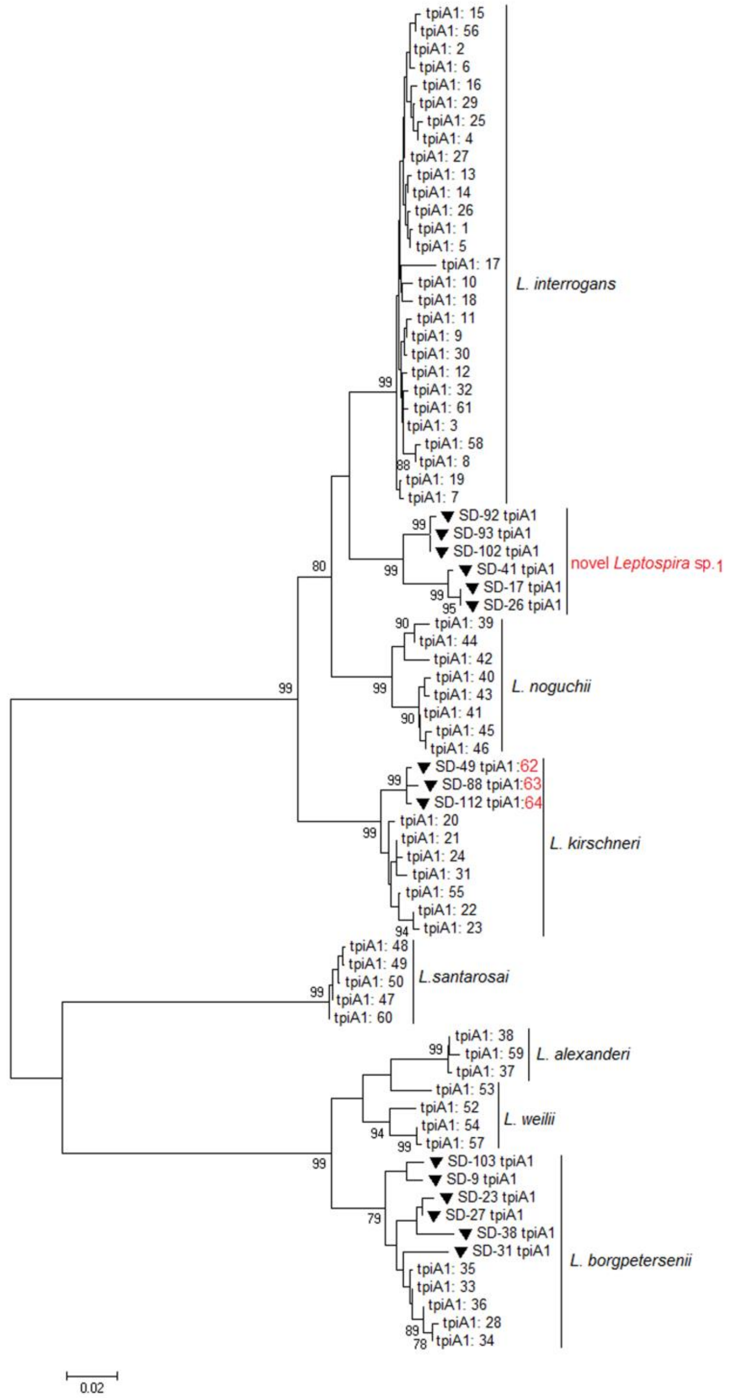

Technical Appendix Figure 4. Neighbor-joining phylogenetic tree for *tpiA* gene of *Leptospira* in bats, Mengyin County, Shandong Province, China. The tree was constructed with Kimura's 2-parameter model using MEGA 7.0, and bootstrap values were calculated with 1,000 replicates. Alleles of each locus detected in this study are shown with the sample number highlighted with a triangle, and novel allele numbers assigned in this study are indicated in red. *Leptospira* species are shown on the right, and potential new *Leptospira* species detected in this study are shown in red.

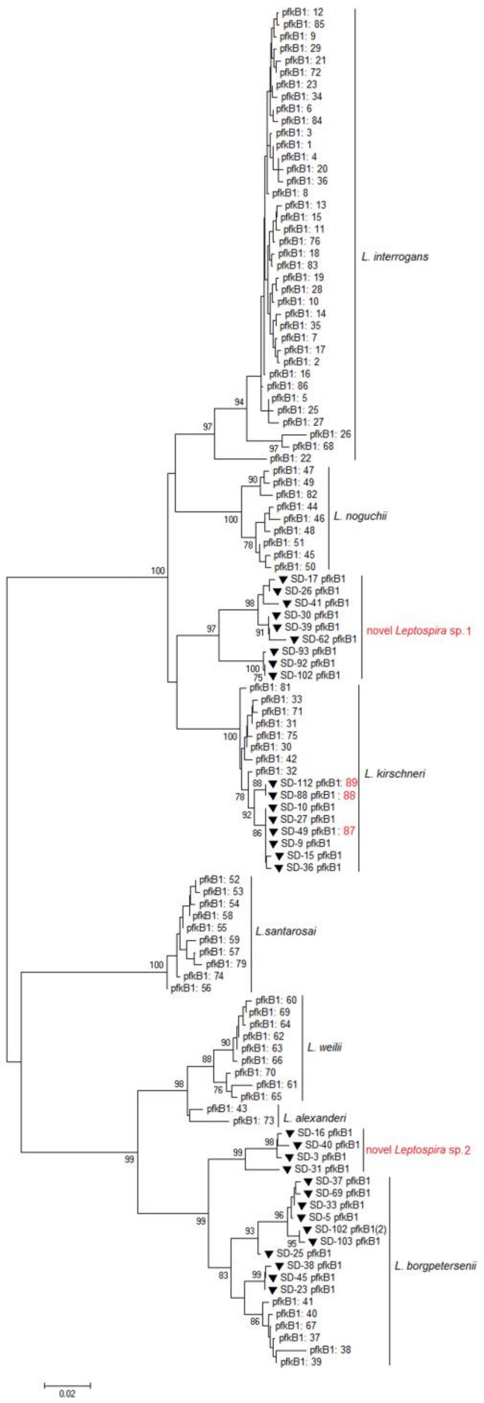

Technical Appendix Figure 5. Neighbor-joining phylogenetic tree for *pfkB* gene of *Leptospira* in bats, Mengyin County, Shandong Province, China. The tree was constructed with Kimura's 2-parameter model using MEGA 7.0, and bootstrap values were calculated with 1,000 replicates. Alleles of each locus detected in this study are shown with the sample number highlighted with a triangle, and novel allele numbers assigned in this study are indicated in red. *Leptospira* species are shown on the right, and potential new *Leptospira* species detected in this study are shown in red.

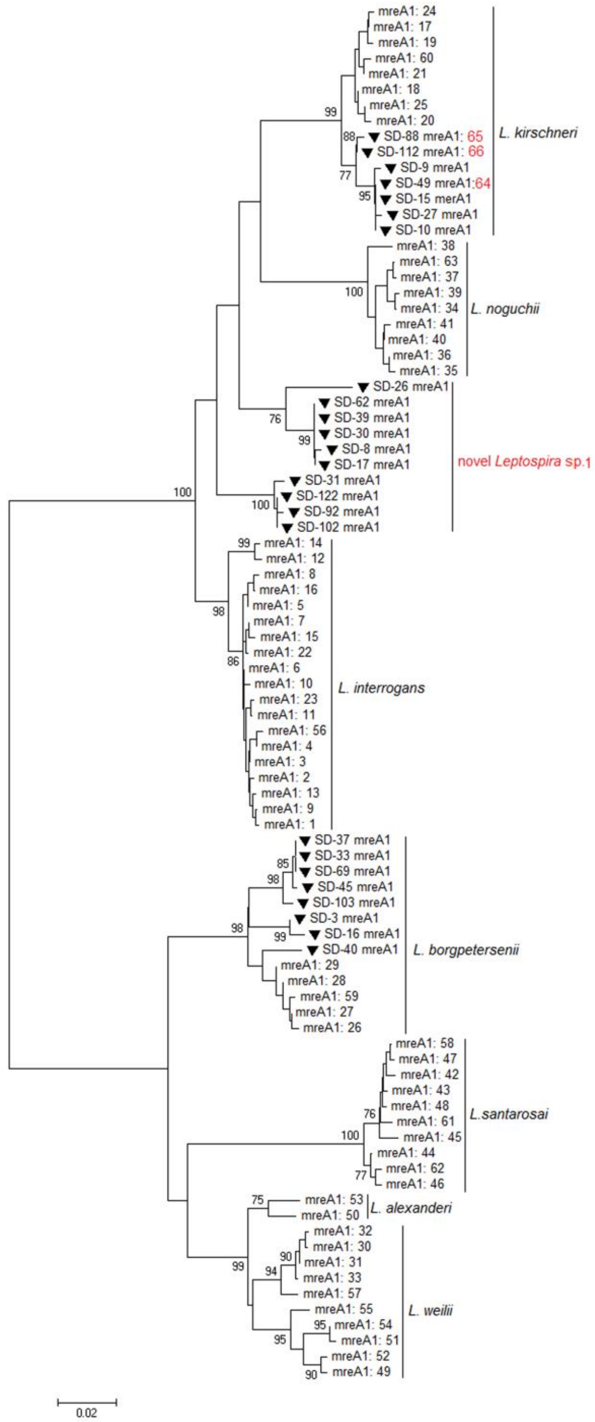

Technical Appendix Figure 6. Neighbor-joining phylogenetic tree for *mreA* gene of *Leptospira* in bats, Mengyin County, Shandong Province, China. The tree was constructed with Kimura's 2-parameter model using MEGA 7.0, and bootstrap values were calculated with 1,000 replicates. Alleles of each locus detected in this study are shown with the sample number highlighted with a triangle, and novel allele numbers assigned in this study are indicated in red. *Leptospira* species are shown on the right, and potential new *Leptospira* species detected in this study are shown in red.

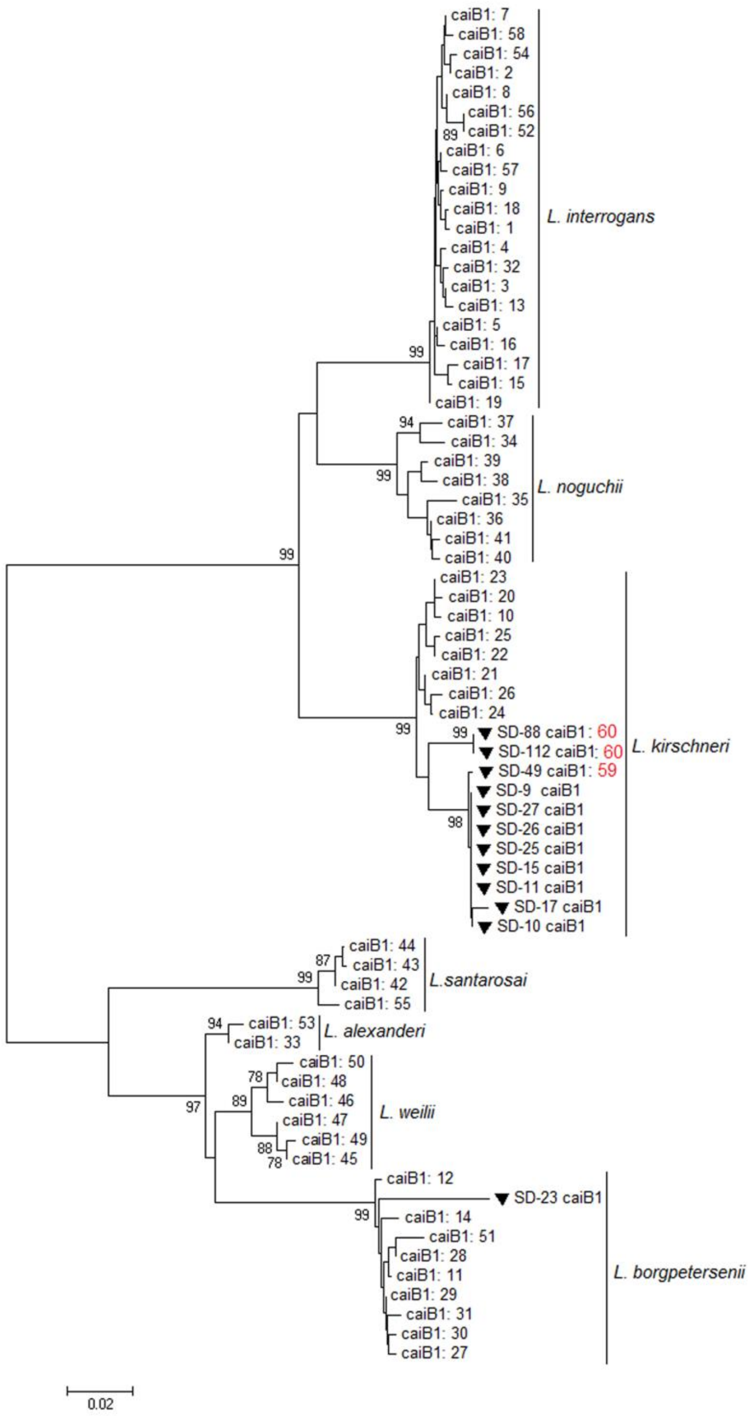

Technical Appendix Figure 7. Neighbor-joining phylogenetic tree for *caiB* gene of *Leptospira* in bats, Mengyin County, Shandong Province, China. The tree was constructed with Kimura's 2-parameter model using MEGA 7.0, and bootstrap values were calculated with 1,000 replicates. Alleles of each locus detected in this study are shown with the sample number highlighted with a triangle, and novel allele numbers assigned in this study are indicated in red. *Leptospira* species are shown on the right, and potential new *Leptospira* species detected in this study are shown in red.
